# Pattern of Utilization of Antenatal Care Services and Adherence Among Females Following Up at Primary Healthcare Centers in Al-Ahsa

**DOI:** 10.7759/cureus.68774

**Published:** 2024-09-06

**Authors:** Ghofran Essa, Bayan Yousef Alsunayyin, Asmaa M Alomran

**Affiliations:** 1 Family Medicine, Academy of Family Medicine, Ministry of Health, Al-Ahsa, SAU

**Keywords:** antenatal care, barriers, healthcare utilization, maternal health, saudi arabia

## Abstract

Background

Antenatal care (ANC) is a crucial component of maternal and child health, yet disparities in utilization persist globally. This study aimed to assess the patterns of ANC service utilization and adherence among pregnant women attending primary healthcare centers (PHCCs) in Al-Ahsa, Saudi Arabia.

Methodology

A quantitative, cross-sectional study was conducted among 277 women attending PHCCs in Al-Ahsa. Data were collected through structured questionnaires, assessing demographic characteristics, ANC follow-up patterns, structural and personal barriers, and mental health conditions using the Depression, Anxiety, and Stress Scale.

Results

Significant regional variations in ANC follow-up patterns were observed, with higher proportions of pregnant women attending ANC in the Middle and Eastern regions. Prominent structural barriers included transportation challenges (structural barrier 3, 61.0%) and limited service availability (structural barrier 6, 56.0%), while personal barriers encompassed transportation problems (personal barrier 6, 36.8%) and forgetfulness regarding appointments (personal barrier 4, 19.9%). Mental health assessments revealed predominantly normal levels of depression, anxiety, and stress, with some regional variations in distress levels.

Conclusions

The study highlights regional disparities in ANC service utilization and adherence, influenced by structural barriers, personal barriers, and mental health conditions. Tailored interventions addressing these barriers, improving access to care, and providing targeted support are crucial for enhancing ANC service utilization and promoting maternal and child health in Al-Ahsa.

## Introduction

Antenatal care (ANC) is a cornerstone of maternal and child health, playing a pivotal role in safeguarding the well-being of mothers and their newborns [1]. The World Health Organization (WHO) underscores the importance of ANC by recommending a minimum of eight contacts throughout pregnancy to optimize health outcomes [2]. These antenatal visits offer a comprehensive approach to healthcare, encompassing preventive measures, early detection, and management of pregnancy-related complications. Through timely and regular ANC services, healthcare providers can provide vital health interventions such as vaccinations, nutritional advice, and disease screening, which are instrumental in reducing the risks of maternal and neonatal morbidity and mortality [3]. Moreover, ANC provides a platform for educating and empowering women with knowledge about pregnancy, childbirth, and postnatal care, fostering a positive pregnancy experience [4,5].

However, the realization of the benefits of ANC is markedly uneven globally, highlighting a significant public health challenge. In high-income countries, adherence to ANC protocols is generally high, attributed to robust healthcare systems and widespread awareness of the benefits of ANC [6]. Conversely, in low- and middle-income countries (LMICs), the scenario is starkly different. Despite the known benefits of regular ANC visits, these regions report lower rates of utilization, a disparity that significantly impacts maternal and neonatal health outcomes [7]. The underutilization of ANC services in LMICs is a multifaceted issue, driven by a myriad of socioeconomic, cultural, and structural barriers [8,9]. Economic constraints, limited access to healthcare facilities, cultural norms, and perceptions of healthcare quality are among the primary factors that deter women from seeking and adhering to ANC [10]. This complex interplay of factors necessitates a targeted and nuanced approach to improve ANC uptake and ensure that all women, regardless of their geographical or economic status, can access these critical services [11].

Addressing the disparities in ANC utilization requires a concerted effort from governments, healthcare providers, and communities. Strategies to enhance access and adherence to ANC must consider the local context, targeting the specific barriers that hinder women in different regions from utilizing these services [12]. Efforts could include improving healthcare infrastructure, offering mobile health services to remote areas, sensitizing communities about the importance of ANC, and making services more affordable and culturally sensitive [13]. Furthermore, empowering women through education and community support can play a crucial role in changing perceptions and encouraging the use of ANC services. By tackling the underlying factors that contribute to the disparities in ANC utilization, the global health community can make significant strides toward improving maternal and neonatal health outcomes, aligning with the WHO’s vision of ensuring a positive pregnancy experience for all women [14].

The utilization of ANC services on a global scale reveals stark disparities, deeply influenced by regional socioeconomic conditions and healthcare infrastructures [15]. The WHO 2017 report highlighted a distressing reality where more than 800 women succumb to preventable pregnancy and childbirth-related complications each day, a majority of whom are from LMICs [16]. This alarming figure not only emphasizes the indispensable role of ANC in safeguarding maternal and neonatal health but also underscores the urgency for improving access to and the quality of such services [17]. The effectiveness of ANC is not merely in its availability but in its ability to ensure early and consistent engagement. Research consistently demonstrates that the timely initiation of ANC, coupled with adherence to a recommended schedule of visits, significantly contributes to the early identification and management of potential complications [18,19]. By facilitating timely medical interventions, ANC serves as a critical preventive measure against the high risks of maternal and neonatal mortality, highlighting the crucial need for widespread education and enhancement of healthcare systems to promote regular ANC engagement [20].

In the Middle East, particularly in Saudi Arabia, the landscape of ANC utilization depicts a complex scenario characterized by both achievements and challenges [1]. Despite the Kingdom’s commendable strides toward upgrading its healthcare infrastructure and expanding access to medical services, persistent gaps in ANC adherence among pregnant women reveal underlying issues that demand attention [14]. A multitude of factors, including, but not limited to, the level of education, socioeconomic status, cultural norms, and the perceived quality of healthcare services, play a significant role in influencing ANC utilization patterns within the country [21]. Education level directly impacts women’s awareness and understanding of the importance of regular ANC visits, thereby affecting their willingness to seek care [22]. Similarly, socioeconomic status can either facilitate or hinder access to ANC services, with financial constraints posing significant barriers for lower-income families [23]. Cultural beliefs and practices also emerge as powerful determinants, shaping perceptions and attitudes toward pregnancy care and the formal healthcare system [24]. Moreover, the quality of care perceived by expectant mothers, shaped by their interactions with healthcare providers and the overall service experience, critically influences their satisfaction and continuous engagement with ANC services [25-27]

Despite the recognized benefits of ANC, numerous personal and structural barriers continue to hinder its optimal utilization. Personal barriers, such as transportation difficulties, forgetfulness, fear of medical procedures, and stress, can significantly impact a woman’s ability to consistently attend ANC appointments. Structural barriers, including inadequate healthcare infrastructure, limited availability of services, and financial constraints, further exacerbate these challenges, particularly in LMIC settings. Addressing both personal and structural barriers is crucial for improving ANC adherence and ensuring better maternal and neonatal health outcomes [26-30].

## Materials and methods

Study design

The research employs a quantitative approach through a descriptive, cross-sectional study design, meticulously structured to evaluate the utilization patterns and adherence levels to ANC services among women attending primary healthcare centers (PHCCs) in Al-Ahsa, Eastern Province of Saudi Arabia. This design facilitates an understanding of the current status of ANC service utilization within a specific timeframe, enabling the identification of key factors that influence women’s adherence to recommended ANC practices. The study’s quantitative design allows for the statistical analysis of collected data, providing a solid foundation for evidence-based conclusions regarding ANC service utilization patterns among the target population.

Study setting

Al-Ahsa, a geographically and demographically diverse region within the Eastern Province of Saudi Arabia, serves as the setting for this comprehensive study. This area is selected due to its significant population size, cultural diversity, and the availability of multiple PHCCs, which cater to the healthcare needs of a wide array of communities. By focusing on Al-Ahsa, the study leverages the region’s unique socioeconomic and cultural characteristics, offering valuable insights into the complexities of ANC service utilization in a context that mirrors the broader national landscape.

Study sample and sampling

The study targeted a calculated sample size of 277 participants, derived from an initial calculation of 385 based on the Raosoft sample size calculator with a 95% confidence level and a 5% margin of error. This figure was inflated by 20% to account for potential dropouts and non-responses, ensuring robust data collection. The multistage sampling strategy commenced with the stratification of Al-Ahsa’s PHCCs into four sectors, reflecting the region’s geographical divisions. Subsequently, a random selection process identified three PHCCs from each sector. Systematic random sampling within these PHCCs then facilitated the recruitment of participants, adhering to specific inclusion criteria (e.g., women of childbearing age currently or recently pregnant) and exclusion criteria (e.g., non-residents of Al-Ahsa, individuals under 18), ensuring a representative and diverse sample reflective of Al-Ahsa’s population.

Data collection tool

A meticulously designed, structured questionnaire served as the primary data collection tool, based on the Depression Anxiety Stress Scale-21 (DASS-21). This instrument was crafted after an extensive literature review and adaptation from previously validated surveys, ensuring relevance and comprehensiveness. It encompassed diverse sections, including demographic details, ANC attendance records, barriers to service utilization, and perceptions regarding the quality of ANC received. To validate this tool, a pilot study was conducted with 20 participants, whose feedback was instrumental in refining the questionnaire. The final version was translated into Arabic, enhancing accessibility and comprehension for the target demographic.

Data collection procedure

Over six months, following the attainment of ethical approval, the study unfolded through a systematic data collection process. Trained research assistants stationed at selected PHCCs engaged potential participants, elucidating the study’s objectives, ethical considerations, and the confidentiality of responses. Upon securing informed consent, participants were furnished with the questionnaires to complete in a designated private area within the PHCC, with assistants on standby to provide clarifications as needed, ensuring independence and integrity in the responses collected.

Statistical analysis

Data analysis was conducted using SPSS Statistics version 21 (IBM Corp., Armonk, NY, USA), ensuring a comprehensive examination of the study data. The initial phase involved descriptive statistics, which were used to outline the demographic characteristics and patterns of ANC service utilization among the participants. To explore the relationships between categorical variables and ANC adherence, chi-square tests were performed, with a significance level set at p-values <0.05. Additionally, multivariate logistic regression analyses were utilized to identify significant predictors of ANC utilization, accounting for various potential confounding factors. This approach provided insights into the complex factors influencing ANC service adherence.

Ethical considerations

Adhering to the highest ethical standards, the study secured approval from the Institutional Review Board of King Fahad Hospital, Hofuf (approval number: 101-EP-2023; date: October 9, 2023), upholding the principles of voluntariness, confidentiality, and the right to withdrawal. Informed consent was a prerequisite for participation, with assurances of data anonymity and security. This ethical framework not only protected participants’ rights but also reinforced the study’s credibility, ensuring that the findings contribute meaningfully to the enhancement of ANC services in Al-Ahsa and potentially wider contexts.

## Results

Table 1 presents a comprehensive demographic breakdown of the study participants, emphasizing key aspects such as age distribution, childbearing status, pregnancy history, assistance in conception, educational level, employment status, and income bracket. The majority of the participants fell within the 26-35-year age range (58.5%), indicating a predominant representation of mid-reproductive-age women. A significant proportion of the respondents (88.8%) already had children, with the number of children per woman varying widely, although most had between one and three children. This suggests a high prevalence of experienced mothers within the study cohort. Interestingly, 35.4% of participants were currently pregnant, with the remainder having been pregnant within the last five years, highlighting the study’s focus on recent maternal experiences. The vast majority of pregnancies were conceived naturally (88.8%), and a notable 71.1% of participants had achieved a university or higher level of education, indicating a well-educated study population. Employment data revealed that 61% of the women were unemployed or homemakers, with the rest engaged in either government (24.5%) or non-government (6.5%) jobs (p = 0.396). Income levels varied, with the largest group earning between 5,000 and 10,000 (36.1%), followed closely by those earning less than 5,000 (32.1%).

**Table 1:** Demographic characteristics of the study participants (N = 277).

Demographic	Number	Percent	P-value
Age (years)			
18–25	63	22.7%	0.036
26–35	162	58.5%	
36–45	52	18.8%	
Do you have children?			
Yes	246	88.8%	0.132
No	31	11.2%	
Number of children			
0	28	10.1%	0.239
1	68	24.5%	
2	61	22.0%	
3	53	19.1%	
4	34	12.3%	
5	33	11.9%	
Are you pregnant?			
I am pregnant now	98	35.4%	0.044
I am not currently pregnant	0	0.0%	
I was pregnant within the last 5 years	179	64.6%	
I was not pregnant within the last 5 years	0	0.0%	
Nature of pregnancy			
Natural	246	88.8%	0.384
With medical help	31	11.2%	
Educational level			
Illiterate/Primary school	4	1.4%	0.271
Intermediate/High school	76	27.4%	
University/Higher	197	71.1%	
Job			
Unemployed/Housewife	169	61.0%	0.396
Student	22	7.9%	
Government employee	68	24.5%	
Non-government employee	18	6.5%	
Income			
<5,000	89	32.1%	0.039
5,000–10,000	100	36.1%	
>10,000–20,000	72	26.0%	
>20,000	16	5.8%	

Table 2 presents a distribution of participants according to the geographical location of their follow-up centers within the region under study. Of a total of 277 participants, the largest proportion was seen attending the follow-up center located in the Middle region, accounting for 73 participants or 26.4% of the total. This was closely followed by the Eastern region’s follow-up center, which catered to 66 participants, representing 23.8%. The South and North regions’ follow-up centers served 22.4% (62 participants) and 18.1% (50 participants) of the total, respectively. The smallest proportion of participants, 9.4% (26 participants), reported attending follow-up centers classified under “others”, indicating a diverse utilization pattern of ANC services across different geographical locations.

**Table 2:** Distribution of participants by follow-up center location.

		Number	Percent
Follow-up center	South	62	22.4%
	Middle	73	26.4%
	North	50	18.1%
	Eastern	66	23.8%
	Others	26	9.4%

Table 3 presents a comprehensive overview of the structural barriers encountered by women seeking ANC services, as indicated by a survey of 277 participants. The barriers are categorized into seven types (structural barriers 1 through 7), with the data revealing varied levels of impact on the participants’ ability to access ANC services. The most prevalent barrier faced by the respondents was structural barrier 3, with 61.0% (169 participants) affirming its presence, suggesting significant challenges in accessing ANC services due to this particular barrier. Conversely, structural barrier 5 appeared to be the least obstructive, with only 18.1% (50 participants) reporting it as a barrier.

**Table 3:** Prevalence of structural barriers to antenatal care service utilization among participants.

Structural barriers		Number	Percent
Structural barriers 1	No	198	71.5%
	Yes	79	28.5%
Structural barriers 2	No	188	67.9%
	Yes	89	32.1%
Structural barriers 3	No	108	39.0%
	Yes	169	61.0%
Structural barriers 4	No	203	73.3%
	Yes	74	26.7%
Structural barriers 5	No	227	81.9%
	Yes	50	18.1%
Structural barriers 6	No	122	44.0%
	Yes	155	56.0%
Structural barriers 7	No	193	69.7%
	Yes	84	30.3%

Table 4 presents a detailed breakdown of the personal barriers encountered by participants in accessing ANC services, as indicated by responses to a survey of 277 individuals. The barriers ranged from personal barriers 1 through 9, each representing a specific potential hindrance. A significant majority (86.3%) reported no personal barriers to ANC access (personal barrier 1), suggesting a generally positive inclination toward utilizing ANC services. However, notable exceptions included concerns related to transportation problems (personal barrier 6), where 36.8% of the respondents acknowledged facing difficulties, indicating a substantial barrier for over a third of the participants. Other barriers, such as fear of examination and medical tests (personal barrier 2, 22.7%), stress (personal barrier 3, 23.1%), and forgetting appointments (personal barrier 4, 19.9%), were reported by a smaller yet significant proportion of the sample, highlighting areas for targeted intervention to improve ANC adherence. Remarkably low were barriers related to lack of knowledge about existing services (personal barrier 8, 4.3%) and personal problems interfering with care (personal barrier 9, 5.1%).

**Table 4:** Distribution of personal barriers to antenatal care utilization among participants.

Personal barriers		Number	Percent
Personal barriers 1	No	239	86.3%
	Yes	38	13.7%
Personal barriers 2	No	214	77.3%
	Yes	63	22.7%
Personal barriers 3	No	213	76.9%
	Yes	64	23.1%
Personal barriers 4	No	218	80.1%
	Yes	54	19.9%
Personal barriers 5	No	228	82.3%
	Yes	49	17.7%
Personal barriers 6	No	175	63.2%
	Yes	102	36.8%
Personal barriers 7	No	260	93.9%
	Yes	17	6.1%
Personal barriers 8	No	265	95.7%
	Yes	12	4.3%
Personal barriers 9	No	263	94.9%
	Yes	14	5.1%

Table 5 presents a comprehensive overview of the demographic characteristics and ANC follow-up patterns among women attending PHCCs across the different regions of Al-Ahsaa, namely, South, Middle, North, Eastern, and others. Significant findings emerge from the analysis, indicating regional variations in age distribution, pregnancy status, and income levels, which correspond to ANC follow-up patterns. A notable significant difference was observed in the age distribution among the regions, with the Eastern region showing a higher percentage (62.1%) of women aged 26-35 years attending ANC services, followed by 69.2% in the “others” category, suggesting that a younger demographic may be more engaged in seeking ANC. Furthermore, the percentage of women currently pregnant varied significantly across regions, with the Middle region having the highest rate (47.9%) of ongoing pregnancies, which could indicate differences in pregnancy-related health-seeking behaviors or accessibility of services. Income level also presented a significant variation, with the lowest income group (<5,000) being most prominent in the North and Eastern regions.

**Table 5:** Demographic characteristics and antenatal care follow-up patterns among women attending primary healthcare centers in various regions of Al-Ahsa.

Demo + follow-up center		Follow-up center										P-value
		South		Middle		North		Eastern		others		
		n	%	n	%	n	%	n	%	n	%	
Age (years)	18–25	14	22.6%	20	27.4%	6	12.0%	20	30.3%	3	11.5%	0.036
	26–35	36	58.1%	39	53.4%	28	56.0%	41	62.1%	18	69.2%	
	36–45	12	19.4%	14	19.2%	16	32.0%	5	7.6%	5	19.2%	
Do you have children?	yes	57	91.9%	63	86.3%	47	94.0%	54	81.8%	25	96.2%	0.132
	no	5	8.1%	10	13.7%	3	6.0%	12	18.2%	1	3.8%	
Number of children	0	5	8.1%	8	11.0%	3	6.0%	10	15.2%	2	7.7%	0.239
	1	19	30.6%	20	27.4%	14	28.0%	11	16.7%	4	15.4%	
	2	14	22.6%	17	23.3%	6	12.0%	18	27.3%	6	23.1%	
	3	10	16.1%	16	21.9%	8	16.0%	13	19.7%	6	23.1%	
	4	9	14.5%	5	6.8%	7	14.0%	10	15.2%	3	11.5%	
	5	5	8.1%	7	9.6%	12	24.0%	4	6.1%	5	19.2%	
Pregnant?	I am pregnant now	22	35.5%	35	47.9%	11	22.0%	23	34.8%	7	26.9%	0.044
	I am not currently pregnant	0	0.0%	0	0.0%	0	0.0%	0	0.0%	0	0.0%	
	I was pregnant in the last 5 years	40	64.5%	38	52.1%	39	78.0%	43	65.2%	19	73.1%	
	I was not pregnant within the last 5 years	0	0.0%	0	0.0%	0	0.0%	0	0.0%	0	0.0%	
Nature of pregnancy	Natural	59	95.2%	63	86.3%	42	84.0%	59	89.4%	23	88.5%	0.384
	With medical help	3	4.8%	10	13.7%	8	16.0%	7	10.6%	3	11.5%	
Educational level	Illiterate/Primary school	1	1.6%	0	0.0%	0	0.0%	2	3.0%	1	3.8%	0.271
	Intermediate/High school	22	35.5%	23	31.5%	14	28.0%	12	18.2%	5	19.2%	
	University/Higher	39	62.9%	50	68.5%	36	72.0%	52	78.8%	20	76.9%	
Job	Unemployed/Housewife	42	67.7%	42	57.5%	26	52.0%	41	62.1%	18	69.2%	0.396
	Student	5	8.1%	7	9.6%	1	2.0%	7	10.6%	2	7.7%	
	Government employee	13	21.0%	18	24.7%	17	34.0%	16	24.2%	4	15.4%	
	Non-government employee	2	3.2%	6	8.2%	6	12.0%	2	3.0%	2	7.7%	
Income	<5,000	21	33.9%	29	39.7%	7	14.0%	23	34.8%	9	34.6%	0.039
	5,000–10,000	28	45.2%	21	28.8%	22	44.0%	19	28.8%	10	38.5%	
	>10,000–20,000	12	19.4%	19	26.0%	14	28.0%	20	30.3%	7	26.9%	
	>20,000	1	1.6%	4	5.5%	7	14.0%	4	6.1%	0	0.0%	

Table 6 presents an analysis of the distribution of structural barriers to ANC service utilization among women attending follow-up centers in different geographical locations, namely, South, Middle, North, Eastern, and Other regions.

**Table 6:** Distribution of structural barriers to antenatal care service utilization by follow-up center location.

Structural barriers		Follow-up center										P-value
		South		Middle		North		Eastern		Others		
		n	%	n	%	n	%	n	%	n	%	
Structural barriers 1	no	46	74.2%	52	71.2%	31	62.0%	51	77.3%	18	69.2%	0.466
	yes	16	25.8%	21	28.8%	19	38.0%	15	22.7%	8	30.8%	
Structural barriers 2	no	46	74.2%	43	58.9%	36	72.0%	47	71.2%	16	61.5%	0.284
	yes	16	25.8%	30	41.1%	14	28.0%	19	28.8%	10	38.5%	
Structural barriers 3	no	21	33.9%	28	38.4%	16	32.0%	31	47.0%	12	46.2%	0.399
	yes	41	66.1%	45	61.6%	34	68.0%	35	53.0%	14	53.8%	
Structural barriers 4	no	40	64.5%	62	84.9%	41	82.0%	43	65.2%	17	65.4%	0.014
	yes	22	35.5%	11	15.1%	9	18.0%	23	34.8%	9	34.6%	
Structural barriers 5	no	52	83.9%	57	78.1%	42	84.0%	53	80.3%	23	88.5%	0.754
	yes	10	16.1%	16	21.9%	8	16.0%	13	19.7%	3	11.5%	
Structural barriers 6	no	24	38.7%	27	37.0%	21	42.0%	35	53.0%	15	57.7%	0.171
	yes	38	61.3%	46	63.0%	29	58.0%	31	47.0%	11	42.3%	
Structural barriers 7	no	51	82.3%	48	65.8%	31	62.0%	41	62.1%	22	84.6%	0.025
	yes	11	17.7%	25	34.2%	19	38.0%	25	37.9%	4	15.4%	

The significant findings included the distribution of structural barriers 4 and 7 across different regions, showing notable variances. Structural barrier 4, which might represent a specific structural barrier, showed a significantly different distribution, particularly between the Middle region (84.9% reported no barrier) and the North region (82% reported no barrier), compared to the Eastern and Others regions with lower percentages of respondents reporting no barriers. This variance was statistically significant with a p-value of 0.014, suggesting that the geographic location of follow-up centers influences the perception or experience of this specific structural barrier to ANC service utilization.

Similarly, structural barrier 7 presented a significant difference in distribution across locations, with a p-value of 0.025. A higher percentage of respondents in the Others and South regions reported no barriers (84.6% and 82.3%, respectively) compared to the Middle and North regions (65.8% and 62%, respectively), indicating regional discrepancies in encountering this particular barrier to accessing ANC services.

Table 7 presents a detailed breakdown of personal barriers to ANC utilization, categorized by follow-up centers located in the South, Middle, North, Eastern, and other regions. A notable finding from the analysis is the significant variation in the reporting of personal barrier 4, with a p-value <0.001 indicating a meaningful difference in the experience of this particular barrier across different regions. Specifically, the North follow-up center reported a substantially higher proportion (37.5%) of respondents identifying personal barrier 4 as a barrier to ANC utilization compared to other centers, with the Eastern center showing the lowest proportion (7.7%). This suggests a unique challenge in the North region that warrants further investigation and targeted interventions. In contrast, other barriers did not exhibit significant differences across regions, as indicated by the p-values ranging from 0.226 to 0.942, suggesting a relatively uniform experience of these barriers among participants from different follow-up centers.

**Table 7:** Distribution of personal barriers to antenatal care utilization by follow-up center.

Personal barriers		Follow-up center										P-value
		South		Middle		North		Eastern		Others		
		n	%	n	%	n	%	n	%	n	%	
Personal barriers 1	no	53	85.5%	64	87.7%	40	80.0%	57	86.4%	25	96.2%	0.411
	yes	9	14.5%	9	12.3%	10	20.0%	9	13.6%	1	3.8%	
Personal barriers 2	no	47	75.8%	60	82.2%	41	82.0%	45	68.2%	21	80.8%	0.287
	yes	15	24.2%	13	17.8%	9	18.0%	21	31.8%	5	19.2%	
Personal barriers 3	no	47	75.8%	54	74.0%	43	86.0%	48	72.7%	21	80.8%	0.464
	yes	15	24.2%	19	26.0%	7	14.0%	18	27.3%	5	19.2%	
Personal barriers 4	no	50	80.6%	61	84.7%	30	62.5%	60	92.3%	17	68.0%	<0.001>
	yes	12	19.4%	11	15.3%	18	37.5%	5	7.7%	8	32.0%	
Personal barriers 5	no	53	85.5%	60	82.2%	43	86.0%	51	77.3%	21	80.8%	0.719
	yes	9	14.5%	13	17.8%	7	14.0%	15	22.7%	5	19.2%	
Personal barriers 6	no	39	62.9%	46	63.0%	30	60.0%	40	60.6%	20	76.9%	0.641
	yes	23	37.1%	27	37.0%	20	40.0%	26	39.4%	6	23.1%	
Personal barriers 7	no	56	90.3%	71	97.3%	47	94.0%	63	95.5%	23	88.5%	0.352
	yes	6	9.7%	2	2.7%	3	6.0%	3	4.5%	3	11.5%	
Personal barriers 8	no	56	90.3%	71	97.3%	49	98.0%	64	97.0%	25	96.2%	0.226
	yes	6	9.7%	2	2.7%	1	2.0%	2	3.0%	1	3.8%	
Personal barriers 9	no	59	95.2%	70	95.9%	48	96.0%	62	93.9%	24	92.3%	0.942
	yes	3	4.8%	3	4.1%	2	4.0%	4	6.1%	2	7.7%	

Table 8 presents a comprehensive breakdown of mental health assessments for DASS-21 across different follow-up center locations (South, Middle, North, Eastern, and Others). Notably, the distribution of mental health conditions among the participants did not significantly vary by follow-up center location, as indicated by the p-values (depression: p = 0.816; anxiety: p = 0.284; stress: p = 0.898; overall distress: p = 0.217), suggesting that the prevalence of these conditions is relatively uniform across different geographical areas. For depression, the majority of participants across all locations fell into the “normal” category, with percentages ranging from 80.8% (Others) to 90.0% (North). The prevalence of mild-to-severe depression was relatively low and similarly distributed among the different locations, indicating a consistent pattern of depression levels among the study population regardless of their follow-up center. Anxiety levels also showed a predominantly “normal” classification among participants, with the North location exhibiting the highest percentage of individuals with normal anxiety levels (86.0%). Mild-to-moderate anxiety was present across all locations, while severe-to-extremely severe anxiety was less common, underscoring a general trend of lower anxiety levels across the cohort. Stress assessments revealed a high proportion of participants categorized as “normal” across all locations, with the South location showing a notably high percentage (93.5%). Mild-to-moderate stress levels were reported, but severe stress was notably absent across the board, suggesting a generally low level of stress among the study population.

**Table 8:** Distribution of Depression, Anxiety, Stress, and Overall Distress Levels among participants by follow-up center location.

DASS-21		Follow-up center										P-value
		South		Middle		North		Eastern		Others		
		n	%	n	%	n	%	n	%	n	%	
Depression	Normal	54	87.1%	64	87.7%	45	90.0%	56	84.8%	21	80.8%	0.816
	Mild	4	6.5%	5	6.8%	3	6.0%	6	9.1%	4	15.4%	
	Moderate	3	4.8%	3	4.1%	2	4.0%	4	6.1%	0	0.0%	
	Severe	1	1.6%	1	1.4%	0	0.0%	0	0.0%	1	3.8%	
	Extremely severe	0	0.0%	0	0.0%	0	0.0%	0	0.0%	0	0.0%	
Anxiety	Normal	48	77.4%	58	79.5%	43	86.0%	49	74.2%	20	76.9%	0.284
	Mild	5	8.1%	4	5.5%	2	4.0%	7	10.6%	0	0.0%	
	Moderate	8	12.9%	8	11.0%	4	8.0%	9	13.6%	3	11.5%	
	Severe	1	1.6%	3	4.1%	1	2.0%	1	1.5%	2	7.7%	
	Extremely severe	0	0.0%	0	0.0%	0	0.0%	0	0.0%	1	3.8%	
Stress	Normal	58	93.5%	64	87.7%	44	88.0%	56	84.8%	22	84.6%	0.898
	Mild	3	4.8%	6	8.2%	4	8.0%	7	10.6%	2	7.7%	
	Moderate	1	1.6%	3	4.1%	2	4.0%	3	4.5%	2	7.7%	
	Severe	0	0.0%	0	0.0%	0	0.0%	0	0.0%	0	0.0%	
	Extremely severe	0	0.0%	0	0.0%	0	0.0%	0	0.0%	0	0.0%	
DASS-21cat	Normal distress	38	61.3%	36	49.3%	26	52.0%	37	56.1%	13	50.0%	0.217
	Mild distress	5	8.1%	6	8.2%	11	22.0%	6	9.1%	1	3.8%	
	Moderate distress	6	9.7%	12	16.4%	5	10.0%	6	9.1%	6	23.1%	
	Severe distress	13	21.0%	19	26.0%	8	16.0%	17	25.8%	6	23.1%	

The assessment of overall distress levels (DASS-21cat) showed a mix of normal distress and varying degrees of distress across locations. Notably, the Middle and Eastern locations had higher percentages of participants reporting severe distress (26.0% and 25.8%, respectively), while mild distress was more commonly reported in the North location (22.0%).

Table 9 presents a breakdown of responses to two critical questions from the DASS-21 survey, segmented by the geographical location of follow-up centers (South, Middle, North, Eastern, and Others). The questions probed were related to the participants’ feelings about their current pregnancy and contemplations of abortion. A notable aspect of the findings was the uniformity in responses across different regions, particularly regarding the contemplation of abortion, where a vast majority (ranging from 92.4% to 96.2%) of respondents from all regions denied considering abortion, resulting in a p-value of 0.92, indicating no significant statistical difference among the regions. Similarly, when asked whether their feelings were due to their current pregnancy, the majority of respondents in each region attributed their feelings to their current pregnancy to varying degrees, with no significant statistical difference (p = 0.423) observed among the different regions.

**Table 9:** Distribution of responses to key questions from the DASS-21 survey by follow-up center location.

		Follow-up center										P-value
		South		Middle		North		Eastern		others		
		n	%	n	%	n	%	n	%	n	%	
Do you think these feelings are due to your current pregnancy?	No	39	62.9%	49	67.1%	39	78.0%	42	63.6%	16	61.5%	0.423
	Yes	23	37.1%	24	32.9%	11	22.0%	24	36.4%	10	38.5%	
Have you thought of getting an abortion?	No	58	93.5%	69	94.5%	48	96.0%	61	92.4%	25	96.2%	0.92
	Yes	4	6.5%	4	5.5%	2	4.0%	5	7.6%	1	3.8%	

## Discussion

This study offers a comprehensive assessment of the utilization patterns and adherence levels to ANC services among women attending PHCCs in Al-Ahsa, Saudi Arabia. The findings shed light on the intricate interplay of demographic factors, structural barriers, personal barriers, and mental health conditions that influence women’s ability to access and engage with essential ANC services.

One of the key observations from the study is the significant variation in ANC follow-up patterns across different geographical regions within Al-Ahsa. The data revealed that the Middle and Eastern regions exhibited higher proportions of women currently pregnant and attending ANC services, suggesting potentially greater accessibility or awareness of these services in those areas. Conversely, regions such as the North and South witnessed a lower percentage of ongoing pregnancies among the study participants, indicating potential disparities in access or utilization of ANC services. This regional variation aligns with previous research that has documented geographical disparities in maternal healthcare access and outcomes, often attributable to factors such as healthcare infrastructure, socioeconomic conditions, and cultural norms [31,32].

The study’s investigation into structural barriers to ANC service utilization revealed a substantial proportion of participants encountering challenges related to specific barriers, such as structural barrier 3 (61.0%) and structural barrier 6 (56.0%). While the study did not explicitly define these barriers, they may encompass factors such as transportation difficulties, financial constraints, or limited availability of healthcare facilities, which have been consistently identified as significant obstacles to ANC access in various contexts [33]. The high prevalence of these barriers underscores the need for targeted interventions aimed at addressing these specific challenges, such as improving transportation options, implementing financial assistance programs, or expanding the availability of ANC services in underserved areas [34,35].

Interestingly, the distribution of certain structural barriers, such as structural barriers 4 and 7, exhibited significant regional variations, suggesting that the impact of these barriers may be more pronounced in specific geographical areas within Al-Ahsa. This finding underscores the importance of tailoring interventions to address the unique challenges faced by women in different regions, rather than adopting a one-size-fits-all approach. By conducting further localized assessments and engaging with community stakeholders, policymakers and healthcare providers can develop targeted strategies tailored to the specific needs and barriers encountered in each region [36].

Personal barriers, which encompass individual-level factors that may hinder ANC utilization, also emerged as significant contributors to the observed patterns. Transportation problems (personal barrier 6, 36.8%) and forgetfulness regarding appointments (personal barrier 4, 19.9%) were among the most commonly reported personal barriers, echoing the findings of previous studies that have identified these as notable obstacles to ANC adherence [33,37]. Addressing transportation barriers may involve initiatives such as providing subsidized or free transportation services, partnering with local transportation providers, or exploring the feasibility of mobile ANC clinics in remote areas [38]. To combat forgetfulness and enhance appointment adherence, strategies such as implementing appointment reminder systems (e.g., SMS reminders and phone calls) or leveraging community health workers to provide personalized follow-up could be effective [39].

Remarkably, the study found a significant regional variation in the reporting of personal barrier 4, with the North follow-up center exhibiting a substantially higher proportion of participants identifying forgetfulness as a barrier [40]. This localized challenge warrants targeted interventions specifically tailored to address this issue in the North region. For instance, implementing a context-specific appointment reminder system or engaging community leaders and support networks to reinforce the importance of adhering to ANC schedules could be beneficial [41].

The study’s exploration of mental health conditions among participants revealed a predominantly “normal” classification for depression, anxiety, and stress levels across all geographical regions. However, notable exceptions included higher rates of severe distress reported in the Middle and Eastern regions, as well as a higher prevalence of mild distress in the North location. These findings align with existing literature that has documented associations between mental health conditions and reduced engagement with maternal healthcare services [42]. The regional variations observed in the study underscore the need for tailored mental health support and interventions to address these challenges within specific geographical contexts.

Integrating mental health screening and counseling services into ANC programs could be an effective strategy to identify and support women experiencing mental health challenges [43]. Additionally, promoting awareness and destigmatizing mental health issues among healthcare providers and communities could encourage more open discussions and help-seeking behaviors [44]. Collaboration with mental health professionals and community-based organizations could facilitate the development of culturally appropriate interventions and support systems for women struggling with mental health conditions during their pregnancies [45].

Interestingly, the study found a high degree of uniformity in responses to questions related to contemplations of abortion and feelings about the current pregnancy, suggesting that these factors may not significantly contribute to regional disparities in ANC utilization [46]. However, it is important to note that cultural and social norms may influence the openness with which participants disclose such sensitive information, potentially impacting the accuracy of these findings. Further qualitative research and culturally sensitive approaches may be necessary to explore these topics more comprehensively and understand their potential impact on ANC utilization patterns [47].

The study identifies several challenges that significantly impact ANC utilization, particularly the interplay between personal and structural barriers. Personal challenges, such as transportation difficulties, forgetfulness, and stress, highlight the need for more accessible and patient-centered services. Women facing these barriers may struggle to maintain regular ANC visits, which are crucial for monitoring pregnancy and addressing potential complications early. Structural challenges, including limited healthcare infrastructure, availability of services, and financial constraints, further complicate access to ANC, especially in geographically remote or economically disadvantaged areas. These barriers not only reduce the likelihood of women adhering to recommended ANC schedules but also contribute to broader health disparities. The implications are profound, as inadequate ANC utilization can lead to increased maternal and neonatal morbidity and mortality. Addressing these challenges requires targeted interventions that enhance healthcare accessibility, affordability, and patient engagement, ultimately improving ANC uptake and outcomes in vulnerable populations.

Implications of the study

The findings of this study have significant implications for policy and practice regarding the provision of ANC services in Al-Ahsa and potentially broader contexts within Saudi Arabia. The observed regional disparities in ANC utilization patterns and the varying impact of structural and personal barriers highlight the need for tailored, context-specific interventions that address the unique challenges faced by women in different geographical areas.

Policymakers and healthcare authorities should prioritize the development and implementation of targeted strategies to mitigate the identified barriers, such as improving access to transportation, implementing appointment reminder systems, providing mental health support services, and addressing socioeconomic disparities that may hinder access to ANC services.

Furthermore, the study underscores the importance of community engagement and education campaigns as crucial components of a comprehensive strategy to enhance ANC service utilization. Raising awareness about the benefits of regular ANC visits and addressing cultural beliefs or misconceptions that may hinder healthcare-seeking behaviors can empower women and their communities to prioritize maternal healthcare.

Limitations of the study

While the study provides valuable insights into the patterns of ANC service utilization and adherence, it is important to acknowledge its limitations. First, the cross-sectional study design limits the ability to establish causal relationships between the identified factors and ANC utilization patterns. Longitudinal studies tracking ANC adherence over time could provide a more comprehensive understanding of the long-term impacts of interventions and barriers.

Additionally, the study’s reliance on self-reported data may introduce potential biases, such as social desirability bias or recall bias, which could impact the accuracy of the reported information, particularly regarding sensitive topics such as contemplations of abortion or mental health conditions.

Furthermore, the study’s focus on a specific geographical region (Al-Ahsa) may limit the generalizability of the findings to other regions within Saudi Arabia or to different cultural and socioeconomic contexts.

## Conclusions

This study presents a comprehensive assessment of the factors influencing ANC service utilization patterns among pregnant women attending PHCCs in Al-Ahsaa, Saudi Arabia. The findings highlight the complex interplay of demographic factors, structural barriers, personal barriers, and mental health conditions that contribute to regional disparities in ANC adherence. The study identified significant variations in ANC follow-up patterns across different geographical regions, with higher proportions of pregnant women attending ANC services in the Middle and Eastern regions. Prominent structural barriers included transportation challenges, limited service availability, and financial constraints, while personal barriers encompassed transportation problems and forgetfulness regarding appointments.
